# Late erosion of Amplatzer Septal Occluder device 9 years after the implantation: a case report

**DOI:** 10.1093/icvts/ivad132

**Published:** 2023-08-17

**Authors:** Nikolaos Koumallos, Costantina Aggeli, Theodoros Milas, Kostantinos Tsioufis

**Affiliations:** Department of Cardiac Surgery, Hippokration General Hospital, Athens, Greece; Cardiology Department, Hippokration General Hospital, Athens, Greece; Department of Cardiac Surgery, Hippokration General Hospital, Athens, Greece; Cardiology Department, Hippokration General Hospital, Athens, Greece

**Keywords:** Cardiac erosion, Amplatzer Septal Occluder, Tamponade

## Abstract

Cardiac erosion is a rare but life-threatening complication after the interventional closure of an atrial septal defect. We present the case of a patient who developed cardiac erosion 9 years after the placement of an Amplatzer Septal Occluder. The patient presented to our hospital with symptoms of tamponade. Surgical exploration revealed a tear in the roof of the left atrium. To our knowledge, this is one of the most delayed presentations reported. In these cases, diagnosis is difficult and a level of clinical suspicion is demanded.

## INTRODUCTION

Interventional closure of atrial septal defects (ASD) with a transcatheter device is the preferred strategy in most patients [1]. Cardiac erosion is a rare but life-threatening complication caused by the protrusion of the edge of the device’s disc into the atrial wall and the aorta. It usually develops in the first few days, but sometimes can occur several months or even years after the procedure [2]. In this article, we present the case of a patient who developed cardiac erosion 9 years after the placement of the device. This is one of the most delayed presentations reported.

## CASE PRESENTATION

**Figure 1:**
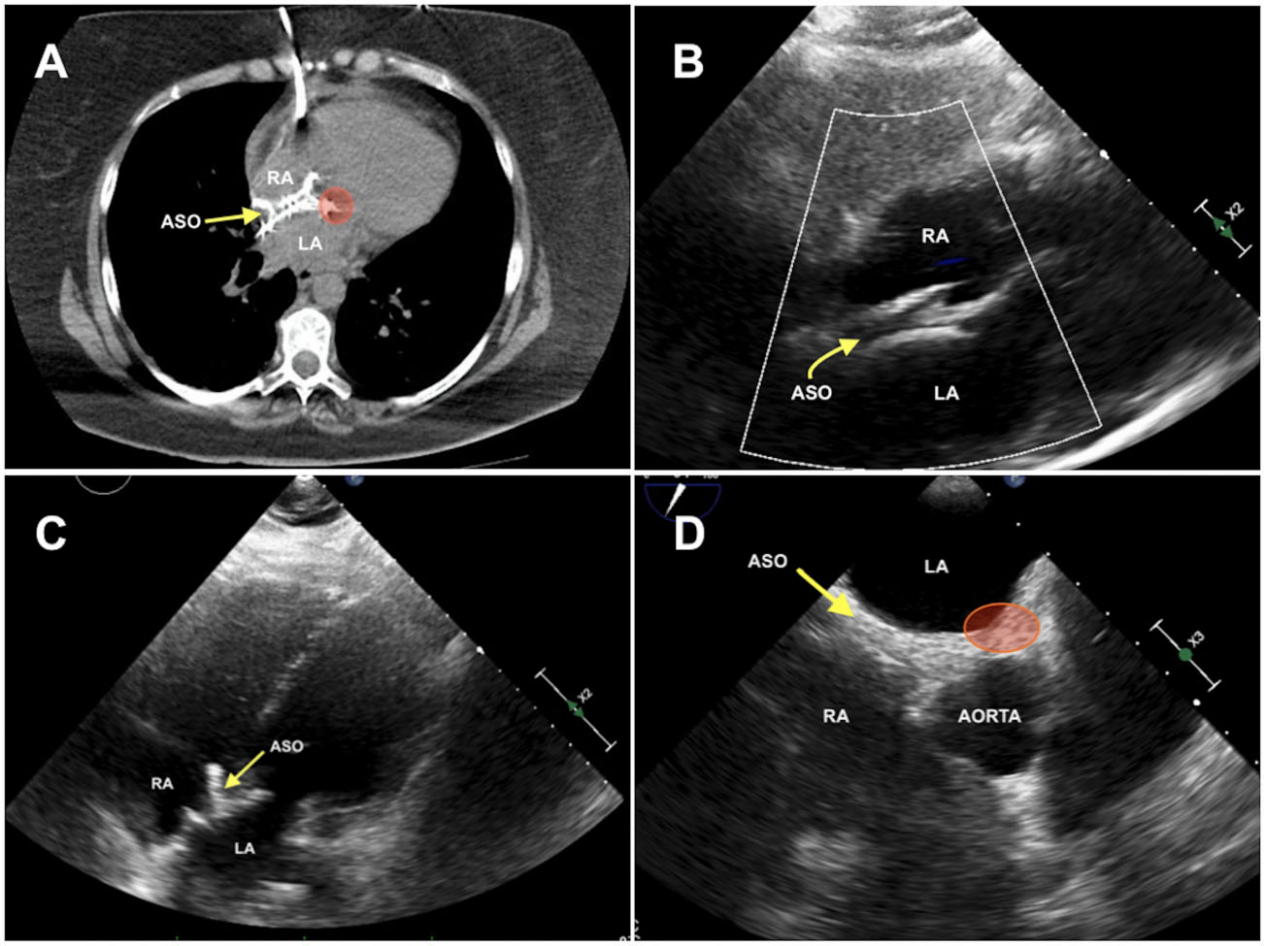
Imaging investigations. The area of injury is demonstrated with a red circle. (**A**) Chest CT scan. (**B**) TTE-subcostal four-chamber view. (**C**) TTE-apical four-chamber view. (**D**) TOE-aortic valve short axis. ASO: Amplatzer Septal Occluder; CT: computed tomography; LA: left atrium; RA: right atrium; TOE: transoesophageal echocardiogram; TTE: transthoracic echocardiogram.

A 50-year-old female presented to the emergency room with symptoms of cardiogenic shock without any previous warning signs. Transthoracic echocardiography revealed a large pericardial effusion with tamponade physiology. Emergency pericardiocentesis was performed and 700 ml of haemorrhagic effusion was drained. After relieving tamponade, the patient was stabilized and referred for further investigation. The patient had atrial septal defect closure 9 years earlier with Amplatzer Septal Occluder (St. Jude Medical, Plymouth, MN) # 38 mm, something that raised the suspicion of cardiac erosion. Both the computer tomography scan and the echocardiograms (transthoracic and transoesophageal) did not give a conclusive diagnosis (Fig. 1 and Videos 1 and 2). Despite the lack of a definite diagnosis, the patient was scheduled for surgery. The decision was driven by the high risk of bleeding recurrence if the device was left in place.

**Figure 2:**
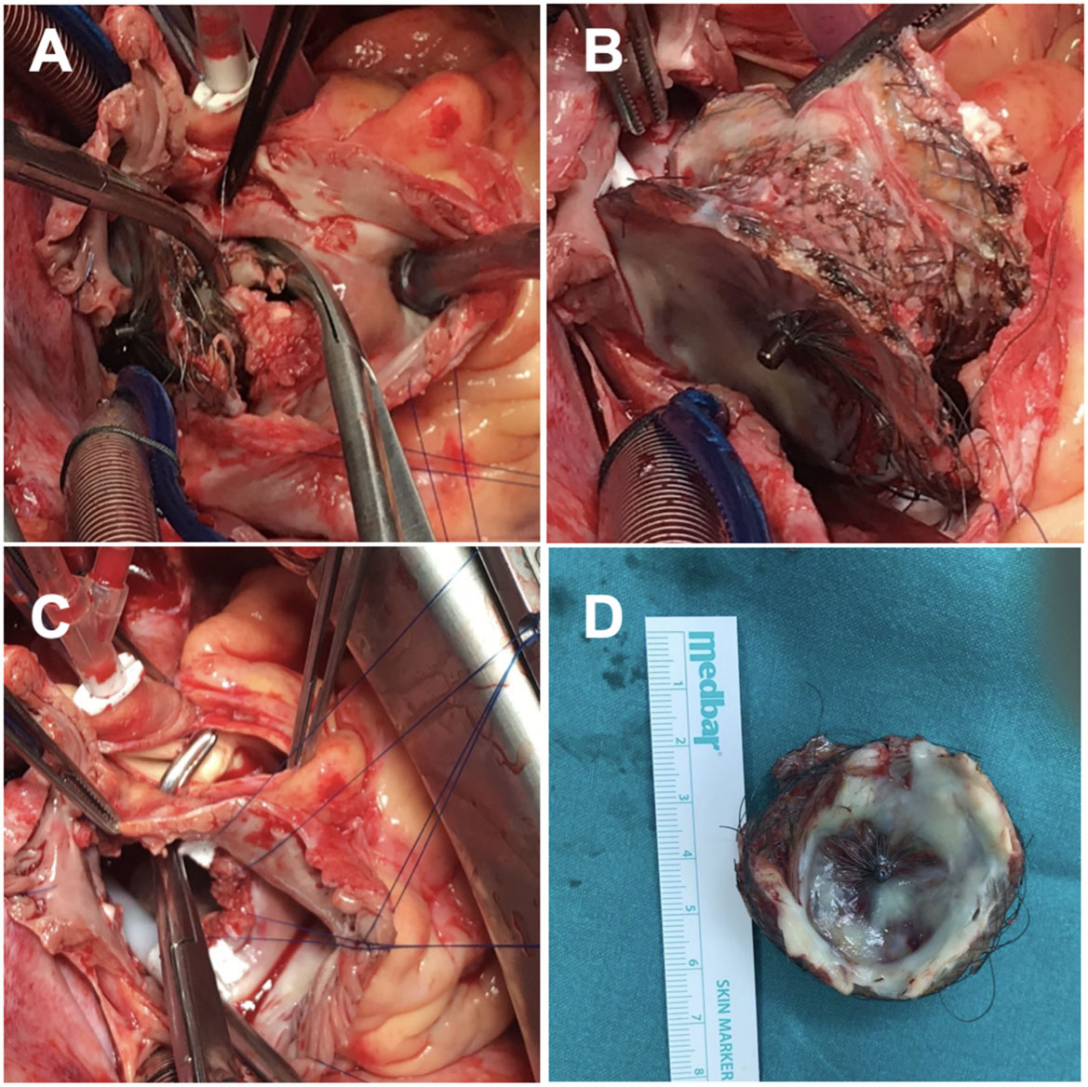
Surgical steps illustrated. (**A**) ASO carefully resected. (**B**) ASO excision. (**C**) Perforation was identified in the roof of the left atrium. (**D**) ASO # 38 mm. ASO: Amplatzer Septal Occluder.

Surgery was performed according to standard protocols. The surgical procedure involved the excision of the occluder through right atriotomy and thorough exploration of the aortic wall and the roof of the right and left atrium (Fig. 2). The perforation was identified in the roof of the left atrium and repaired with interrupted pledgeted 3–0 polypropylene. For the ASD repair, a larger than usual pericardial patch was required. The lack of a sufficient septal rim can make the procedure particularly challenging. Therefore, while resecting the occluder great care was taken to ensure an appropriate septal rim. The patient had an uncomplicated postoperative course and was discharged from the hospital on postoperative day 7.

## DISCUSSION

The Amplatzer Septal Occluder is considered safe and effective [1]. The incidence of cardiac erosion is in the region of 0.1–0.3%. However, the mortality rate was estimated to be as high as 20% [2]. McElhinney *et al.* [3] reported that only 6% of cardiac erosions occur more than five years after implantation. In our case, the complication developed 9 years after the procedure.

A number of risk factors have been related to this complication. Amin *et al.* [4] reported that over 90% of cardiac erosions occur in patients with aortic rim deficiency. It has also been related to superior rim deficiency and placement of an oversized device [5]. In our case, the maximum ASD diameter was 29 mm and the size of the occluder was the largest available (# 38). The aortic rim was absent while the minimum superior rim was 6.8 mm, the posterior rim was 7.8 mm and the inferior vena cava rim was 7.9 mm. Therefore, we believe that ASD on this occasion should have been treated surgically in the first place.

Cardiac tamponade is the most common clinical manifestation of cardiac erosion. The diagnosis is difficult and the decision for surgical exploration is challenging. In our patient, both computer tomography scan and echocardiograms were inconclusive and our decision was driven by the high clinical suspicion and the absence of any other obvious cause of the haemorrhagic effusion.

## CONCLUSION

Diagnosis and treatment of cardiac erosion are not always easy. It may occur even a long time after the placement of the device and in these cases, a level of clinical suspicion is demanded. The surgical repair may also be extremely challenging. On the other hand, cardiologists should consider referring patients with ASD for surgery in the first place when a number of risk factors are present.

**Conflict of interest: **none declared.

## Reviewer information

Interdisciplinary CardioVascular and Thoracic Surgery thanks Ihsan Bakir, Giuseppe D'Ancona and the other anonymous reviewer(s) for their contribution to the peer review process of this article.
